# Metabolic engineering of *Caldicellulosiruptor bescii* for hydrogen production

**DOI:** 10.1007/s00253-023-12974-7

**Published:** 2024-01-09

**Authors:** Minseok Cha, Jung Kon Kim, Won-Heong Lee, Hyoungwoon Song, Tae-Gi Lee, Sun-Ki Kim, Soo-Jung Kim

**Affiliations:** 1 Research Center for Biological Cybernetics, Chonnam National University, Gwangju, 61186 Republic of Korea; 2 Department of Animal Environment, National Institute of Animal Science, Wanju, 55365 Republic of Korea; 3 Department of Integrative Food, Bioscience and Biotechnology, Chonnam National University, Gwangju, 61186 Republic of Korea; 4 Institute for Advanced Engineering, Gyeonggi, 17180 Korea; 5 Department of Food Science and Biotechnology, Chung-Ang University, Gyeonggi, 17546 Republic of Korea

**Keywords:** Hydrogen, Lignocellulosic biomass, *Caldicellulosiruptor bescii*, Consolidated bioprocessing (CBP), Metabolic engineering

## Abstract

**Abstract:**

Hydrogen is an alternative fuel for transportation vehicles because it is clean, sustainable, and highly flammable. However, the production of hydrogen from lignocellulosic biomass by microorganisms presents challenges. This microbial process involves multiple complex steps, including thermal, chemical, and mechanical treatment of biomass to remove hemicellulose and lignin, as well as enzymatic hydrolysis to solubilize the plant cell walls. These steps not only incur costs but also result in the production of toxic hydrolysates, which inhibit microbial growth. A hyper-thermophilic bacterium of *Caldicellulosiruptor bescii* can produce hydrogen by decomposing and fermenting plant biomass without the need for conventional pretreatment. It is considered as a consolidated bioprocessing (CBP) microorganism. This review summarizes the basic scientific knowledge and hydrogen-producing capacity of *C. bescii*. Its genetic system and metabolic engineering strategies to improve hydrogen production are also discussed.

**Key points:**

*• Hydrogen is an alternative and eco-friendly fuel.*

*• Caldicellulosiruptor bescii produces hydrogen with a high yield in nature.*

*• Metabolic engineering can make C. bescii to improve hydrogen production.*

## Introduction

Currently, there are increasing concerns about serious environmental problems such as the greenhouse effect, global climate change, fine dust caused by the use of fossil fuels, and other complications caused by the depletion of fossil fuels (Fawzy et al. 2020; Manisalidis et al. 2020; Martins et al. 2019). To address these issues, there has been a growing interest in biofuels, such as bioethanol, biodiesel, and biohydrogen, which are produced through biological processes using various renewable resources (Cha et al. 2013a; Hoang et al. 2023; Jeswani et al. 2020; Martínez-Jaramillo et al. 2019). Among these biofuels, hydrogen is an attractive and promising option for two important reasons: (i) hydrogen is non-toxic and does not release the greenhouse gas CO_2_ when combusted (clean energy), and (ii) it carries higher energy compared to other hydrocarbon fuels (Hassan et al. 2023; Okolie et al. 2021). Therefore, hydrogen has been suggested as a major chemical energy carrier and could be utilized as a high-energy storage for transportation vehicles (Miller et al. 2021). Hydrogen can be utilized not only as a commercial transportation fuel but also in the chemical industry (chemical looping hydrogen) for the production of methanol and ammonia (Palone et al. 2023), as well as in various other industries such as electronics, metals, and food. Unfortunately, hydrogen does not exist in a free form in nature. However, it is present in water or in the main components of all living organisms, suggesting that hydrogen can be produced through biological processes (Akhlaghi and Najafpour-Darzi 2020; Lepage et al. 2021).

**Fig. 1:**
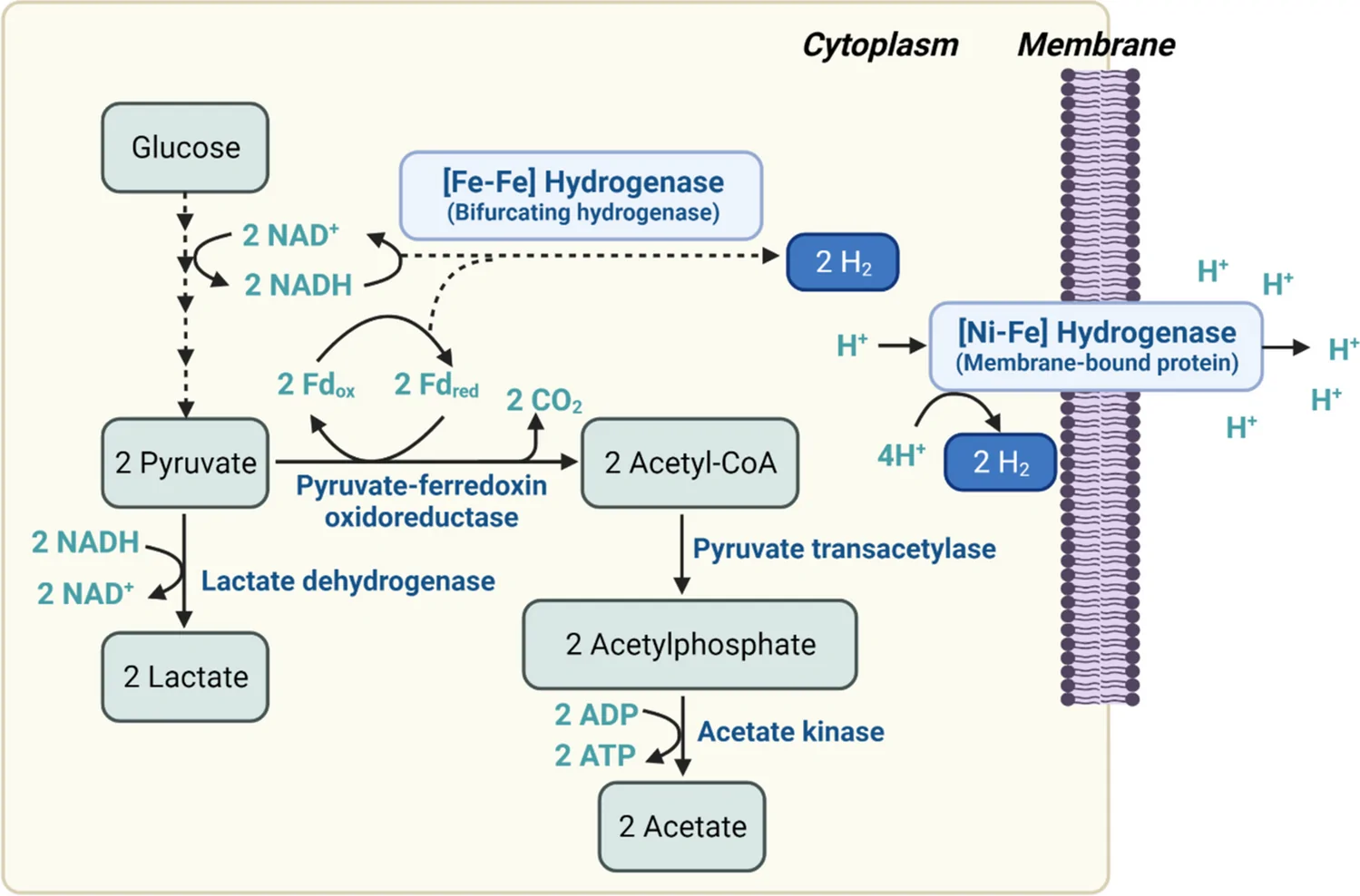
A predicted and simplified biosynthetic pathway for hydrogen production in *C. bescii*

The biological production of hydrogen can be accomplished through various steps including enzymatic saccharification of renewable biomass to convert into fermentable sugars, as well as anaerobic fermentation of these sugars to hydrogen by anaerobic bacteria (Fig. 1) (Alicia Benitez et al. 2021; Cha et al. 2013a, 2016). C5 and C6 sugars, which are derived from a variety of carbohydrates like glucose and xylose found in plant biomass, are oxidized via the Embden–Meyerhof–Parnas glycolytic pathway (Fig. 1) to produce acetate, lactate, carbon dioxide, and hydrogen (Cha et al. 2013a, 2016, 2023; Chandel 2021). In terms of final fermentative products, carbon flow is directed towards lactate or acetyl-CoA, while electrons flux towards lactate and H_2_ from pyruvate, which serves as a major metabolic branch point (Fig. 1).

Microorganisms are crucial for achieving high yields of hydrogen, especially with the involvement of thermophiles such as *Thermoanaerobacter tengcongensis* (~ 4.0 mol H_2_/mol glucose) (Soboh et al. 2004), *Thermotoga maritima* (~ 4.0 mol H_2_/mol glucose) (Schroder et al. 1994; Singh et al. 2019)*,* and *Thermococcus kodakarensis* (~ 3.3 mol H_2_/mol glucose) (Burkhart et al. 2019; Kanai et al. 2005). The hyperthermophile, *Pyrococcus furiosus* (optimal temperature 90 °C)*,* also produced ~ 2.8 mol H_2_/mol glucose (Servé and Kengen 1994; Song et al. 2019) although it is smaller compared to others. The utilization of high temperatures (> 50 °C) should be beneficial for hydrogen production due to reduced viscosity, improved mixing, trace contamination, enhanced reaction rates, and the elimination of the need for reactor cooling (Shahbeik et al. 2022). Additional microorganisms capable of producing hydrogen are listed in Table 1. While most hydrogen-producing microorganisms rely on starch-based biomass, which can be easily saccharified, the direct production of hydrogen from lignocellulosic biomass is challenging for microorganisms as it requires additional steps, such as pretreatment, enzymatic saccharification, and the generation of fermentation inhibitors (Zafar et al. 2021). Therefore, the development of microbial strains that fermenting lignocellulosic biomass effectively is necessary (Kim et al. 2022).

**Table 1:** Hydrogen production by various microorganisms

Microorganisms	Optimal growth temp. (°C)	*Y*_*H/G*_ (mol/mol)*	Final products (except for H_2_ and CO_2_)	References
*Clostridium acetobutylicum* ATCC824	30	1.79	Acetate, acetone, butanol, butyrate	Oh et al. (2009)
*Clostridium beijerinckii*	37	2.81	––-	Lin et al. (2007)
*Clostridium butyricum*	37	2.29	––-	Lin et al. (2007)
*Clostridium thermosaccharolyticum* LMG 6564	55	1.6	Acetate, lactate, ethanol, butanol, butyrate	Vancanneyt et al. (1990)
*Clostridium thermocellum* 27,405	60	1.6	Acetate, lactate, ethanol, formate	David et al. (2006)
*Caldicellulosiruptor saccharolyticus*	70	2.5	Acetate, lactate	de Vrije et al. (2007)
*Caldicellulosiruptor bescii* JWCB001	75	1.8	Acetate, lactate	Cha et al. (2013a)
*Escherichia coli*	37	1.4	Ethanol, acetate, lactate, succinate	Seppälä et al. (2011)
*Escherichia coli* MG1655	37	0.56	Acetate, lactate	Cofré et al. (2016)
*Enterobacter aerogenes*	37–55	1.92	Acetate, lactate, ethanol	Jayasinghearachchi et al. (2009)
*Enterobacter aerogenes E.82005*	38	1.0	Acetate, lactate, ethanol	Tanisho (1998)
*Klebsiella oxytoca*	38	1.0	––-	Minnan et al. (2005)
*Klebsiella pneumoniae*	37	2.7	Acetate, lactate, formate, 2,3-butanediol, ethanol	Niu et al. (2010)
*Thermoanaerobacter tengcongensis* JCM 11007	75	4.0	Acetate	Soboh et al. (2004)
*Thermotoga maritima* DSM 3109	80	4.0	Acetate	Schroder et al. (1994)
*Thermococcus kodakarensis* KOD1	85	3.3	Acetate, alanine	Kanai et al. (2005)
*Thermotoga neapolitana*	77	2.4	––-	de Vrije et al. (2010)
*Thermoanaerobacterium thermosaccharolyticum*	55–60	1.8	Ethanol, D-/L-lactate, acetate	Liu et al. (2008)
*Pyrococcus furiosus* DSM 3638	90	2.8	Acetate, alanine	Servé and Kengen (1994)

**Fig. 2:**
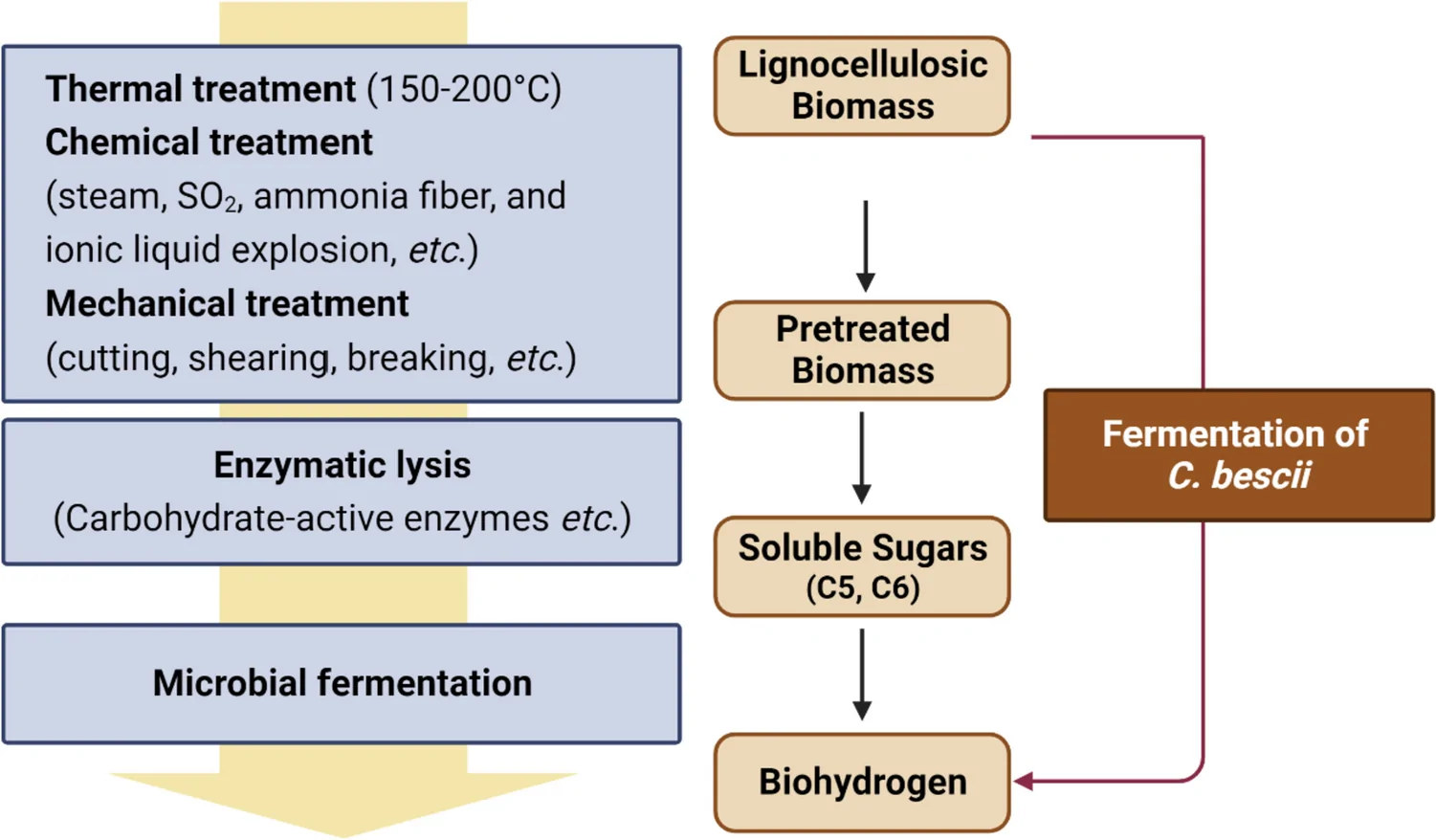
Hydrogen production with no or reduced pretreatment by a CBP (consolidated bioprocessing) organism, *Caldicellulosiruptor bescii*

The genus *Caldicellulosiruptor* is a thermophilic microorganism with cellulosic activity. It can produce hydrogen at high rates from lignocellulosic biomass, with an optimal growth temperature between 75 and 80 °C (Scott et al. 2019). *C. bescii* can serve as a consolidated bioprocessing (CBP) organism (Fig. 2) because it can utilize both C5 and C6 sugars simultaneously and directly convert lignocellulosic biomass without conventional pretreatment steps (Fig. 2) (Cha et al. 2013a, 2016; Chung et al. 2014; Periyasamy et al. 2023). The *C. bescii* genome encodes many carbohydrate-active enzymes (CAZymes), which are multi-domain enzymes with cellulolytic and hemicellulolytic activity and utilize a broad range of substrates, including plant biomass, without the need for conventional pretreatment (Kim et al. 2019). Therefore, there is potential to improve the economics of biofuel production from lignocellulosic biomass by skipping thermal, chemical, and mechanical treatment steps (Cha et al. 2013a; Chung et al. 2014).

This review provides the scientific knowledge and data on *C. bescii*, focusing on its H_2_ production capacity, genetic system, and metabolic engineering strategies, which can make *C. bescii* a highly efficient organism for hydrogen production at high temperatures.

### Biosynthetic pathway of hydrogen of *C. bescii*

Usually, the genus *Caldicellulosiruptor* produces relatively high yields of H_2_ (4 mol of H_2_/ mol of glucose) compared with other microorganisms (Cha et al. 2013a, 2016; Straub et al. 2020). Additionally, acetate should be coupled with H_2_ production for the reoxidation of NADH (a two-electron donor) and ferredoxin (a one-electron donor) (Buckel 2021; Cha et al. 2016; White 2012) (Fig. 1).

As shown in Fig. 1, H2 is produced through proton reduction catalyzed by hydrogenases (Cha et al. 2016; Jay et al.^ 2020^; Lu and Koo 2019). These hydrogenases are metalloenzymes that contain iron in their active site, such as di-iron, nickel–iron, or iron-sulfur clusters (Lu and Koo 2019). Specifically, *Caldicellulosiruptor* spp. have only two types of hydrogenases: bifurcating [Fe–Fe] hydrogenase and [Ni–Fe] hydrogenase (Cha et al. 2013a, 2016; Zhang et al. 2021). NADH and ferredoxin are catabolized by the bifurcating [Fe–Fe] hydrogenase, resulting in the production of H_2_ (Cha et al. 2016; Zhang et al. 2021). On the other hand, the [Ni–Fe] hydrogenase is a membrane-bound heterodimer and is widely found in nature (Alfano and Cavazza 2020). Although the [Ni–Fe] hydrogenase also catalyzes H_2_ production, the bifurcating [Fe–Fe] hydrogenase is the primary enzyme for H_2_ production in *C. bescii,* while the main role of the [Ni–Fe] hydrogenase is to pump out protons across the cellular membrane to generate the “proton motive force” (Kaila and Wikstrom 2021; White 2012).

### Hydrogen production from C5 and C6 sugars

In most of the studies reported, the maximum amount of hydrogen produced was 2.0–3.8 mol of H_2_ /mol of glucose (C6 sugars) due to the formation of co-products such as lactic acid and acetic acid (Esercizio et al. 2021). The theoretical molar yield of hydrogen from xylose (C5 sugar) fermentation is 3.3 mol of H_2_/mol of xylose (C5 sugar) with acetate as the sole byproduct, but the reported values were lower than 2 mol of H_2_/mol of xylose (Chiu-Yue Lin and Hung 2008). Hydrogen production has been reported to be between 0.5 and 4 L/L/day (Ghimire et al. 2015; Beckers et al. 2015). The nature, carbohydrate content, and biodegradability of carbon substrate play an important role in the H_2_ yield, production rate, and overall economics of the process (Nanqi et al. 2011). Many bacterial species have been reported to produce hydrogen from C5 and C6 sugars, including enteric bacteria such as *Enterobacter aerogenes*, *Enterobacter cloacae*, and *Escherichia coli*, which produce about 1–2 mol of H_2_/mol of glucose (Yoshida et al. 2006). *Clostridium* spp. also produce similar amounts (Liu et al. 2006). *Caldicellulosiruptor* spp. produce about 3–4 mol of H_2_/mol of glucose (Willquist et al. 2010). *Enterobacter* utilizes a formate-H_2_ lyase, and *Clostridium* spp. use a ferredoxin-dependent hydrogenase to avoid the thermodynamically unfavorable formation of H_2_ from NADH (Schut and Adams 2009). Based on previous studies on various microorganisms, metabolic engineering and pre-treatment of substrates can increase hydrogen production by improving the biodegradability of substrates. In summary, *C. bescii* can be a superior organism as it does not contain competing pathways other than lactate, offers the potential to produce maximum amounts of H_2_ (4 mol of H_2_/mol of C5 and C6 sugars), and is tractable to metabolic engineering.

### Hydrogen production from various biomass by *C. bescii*

The most important aspect of *C. bescii* is its ability to decompose various monosaccharides and polysaccharides, such as glucose, xylose, crystalline cellulose, and non-pretreated plant biomass. To compare hydrogen production from real-world substrates, *C. bescii* wild type (JWCB001) and its mutant strains (JWCB005 and 017) were grown on 0.5% switchgrass (Cha et al. 2013a). The strains were incubated for 120 h; then, hydrogen production was measured. The hydrogen productions of JWCB001 and JWCB005 were ~ 1.8 mol/mol of glucose and ~ 1.7 mol/mol of glucose, and it is a bit lower than H_2_ production by *Caldicellulosiruptor saccharolyticus* (~ 2.5 mol/mol of glucose). However, in the case of *C. saccharolyticus*, yeast extracts were added to the growth medium. Even without the addition of yeast extracts, the engineered *C. bescii* strain JWCB017 produced significantly more hydrogen (~ 3.4 mol/mol of glucose; Table 2) (Cha et al. 2013a). Actually, *C. bescii* lacks the enzyme required for ethanol production. Chung et al*.* reported the heterologous expression of *adh*E to enable *C. bescii* to produce alcohol (Chung et al. 2014). However, this resulted in reduced hydrogen production as carbon and electrons were redirected for alcohol production (Chung et al. 2014). *C. bescii* can also produce hydrogen from barley straw and *Miscanthus*. In a previous study, JWCB018 without the chromosomal *ldh* gene produced 63% and 25% more hydrogen from barley straw and *Miscanthus* than the wild-type strain (JWCB001), respectively. It might be due to a decrease in lactate production by interrupting lactate dehydrogenase function by a native active transposon (Cha et al. 2023, 2013b). Yilmazel and Duran reported hydrogen production in co-substrate reactors, where *C. bescii* was grown on four different substrate mixtures of cattle manure (CM), switchgrass (SG), and biosolid (BS). *C. bescii* grown on BS + SG + CM (~ 15.0 mM) showed much better hydrogen production compared to BS + SG (~ 11.0 mM), exhibiting synergistic effects of co-fermentation of these feedstocks (Yilmazel and Duran 2021).

**Table 2:** Hydrogen production from biomass by *C. bescii*

Strain	Substrate	Hydrogen produced	References
*C. bescii* DSM 6725 (wild type)	CM + SG	~ 11.5 mM	Yilmazel and Duran (2021)
*C. bescii* DSM 6725 (wild type)	CM + BS	~ 13.0 mM	
*C. bescii* DSM 6725 (wild type)	SG + BS	~ 11.0 mM	
*C. bescii* DSM 6725 (wild type)	CM + SG + BS	~ 15.0 mM	
*C. bescii* DSM 6725 (wild type)	0.5% SG	~ 1.8 mol/mol of glucose	Cha et al. (2013a)
*C. bescii* JWCB005 (*Δpyr*AF)	0.5% SG	~ 1.7 mol/mol of glucose	
*C. bescii* JWCB017 (*Δpyr*AF *Δldh*)	0.5% SG	~ 3.4 mol/mol of glucose	
*C. bescii* JWCB018 (*Δpyr*AF *Δldh ΔcbeI*)	1% Barley Straw	~ 12.0 mM	Cha et al. (2023)
*C. bescii* JWCB018 (*Δpyr*AF *Δldh ΔcbeI*)	1% Miscanthus	~ 17.0 mM	
*C. bescii* JWCB018* (ΔpyrAF Δldh ΔcbeI)*	2% SG	14.5 mM	Chung et al. (2014)
*C. bescii* JWBC032 (*ΔpyrAF ldh::ISCbe4 Δcbe1::PS-layer Cthe-adhE2/(ura-/5-FOA*^*R*^)	2% SG	9.8 mM	
*C. bescii* JWCB038 (*ΔpyrFA Δldh CIS1::PS-layer Cthe-adhE ΔhypADFCDE/(ura-/5-FOA*^*R*^)	2% SG	~ 4.0 mM	Cha et al. (2016)

*CM* cattle manure, *SG* switchgrass, *BS* biosolids

### Genetic system for *C. bescii*

**Fig. 3:**
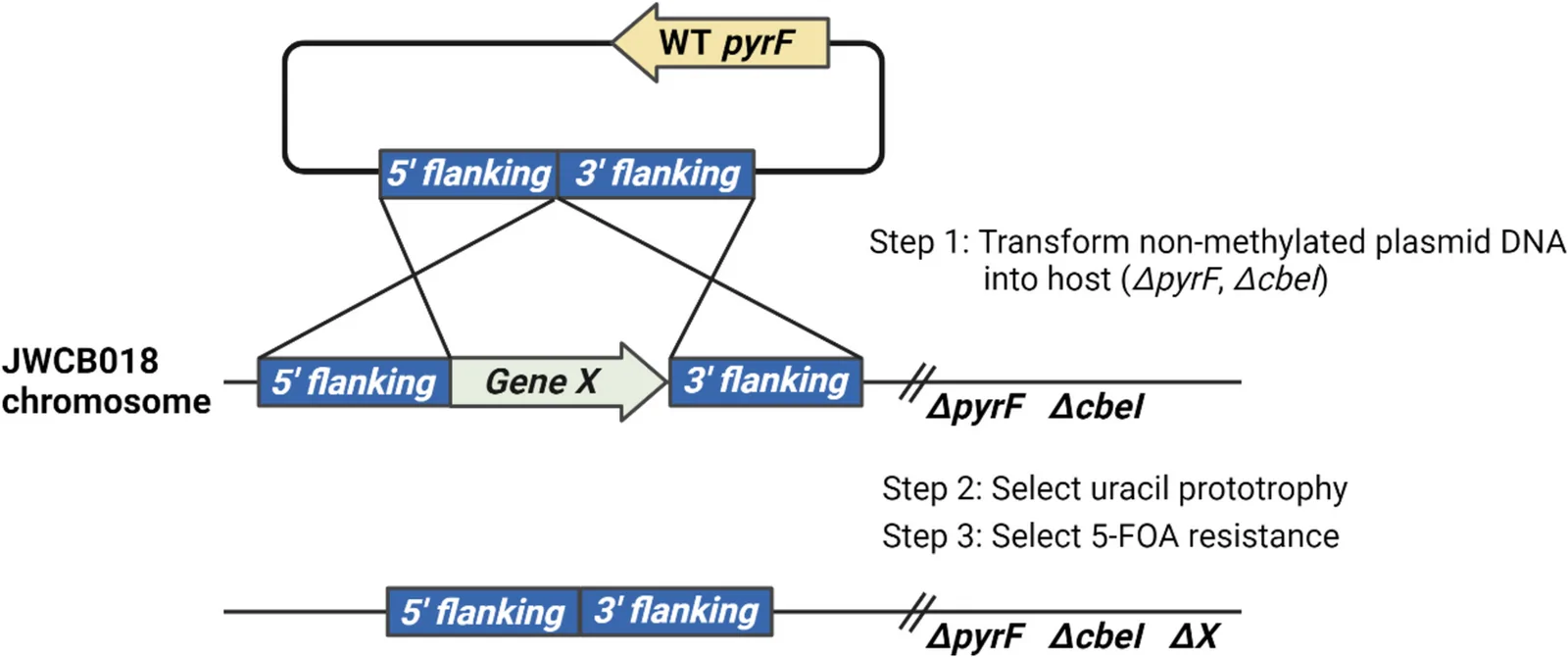
A procedure for editing a target gene in the genome of *C. bescii*

There are many interesting thermophiles that produce interesting and important chemicals. The ability to manipulate *C. bescii* genes is required to make the hyper-thermophilic strain more useful in the real world. However, the wild-type strain of *C. bescii* is not sufficient to produce biofuel, so it needs to be metabolically engineered to produce biofuel at a suitable yield. One of the most difficult aspects of studying hyperthermophiles like *C. bescii* is the lack of genetic tools for metabolic engineering. In order to develop a genetic tool, there are several requirements: (i) overcoming the restriction-modification (R-M) system, (ii) constructing a *E. coli-C. bescii* shuttle vector, (iii) establishing a transformation method, and (iv) selecting a selection marker (Chung et al. 2013a). One significant barrier to develop genetic tools for uncharacterized microorganisms, especially hyperthermophiles, is the lack of selectable markers. Antibiotics are typically used in mesophilic bacteria, but not in thermophiles because thermostable antibiotic markers are usually not available at high temperatures over 70 °C (Crosby et al. 2019). Because of their high growth temperatures, the genetics of most thermophiles depend on auxotrophic mutant strains. This method is often used for many thermophiles including *Caldicellulosiruptor* (Cha et al. 2013a, 2016; Chung et al. 2014; Lipscomb et al. 2016), *Sulfolobus* (Wagner et al. 2012; Zheng et al. 2012), and *Thermotoga* sp. RQ7 (Han and Xu 2017). The selection method (using an auxotroph mutant strain) for transformation in *C. bescii* relies on the loss of the uracil biosynthetic enzyme coding for orotidine-5′-monophosphate (OMP) decarboxylase (*pyr*F), which was first described in yeast (Boeke et al. 1984) and has been a useful genetic tool in both bacteria and archaea (Lucas et al. 2002). In order to generate a spontaneous *pyr*F mutant strain, the cells were grown on low osmolarity-defined growth medium (LOD) (Farkas et al. 2013) supplemented with uracil and 5-fluoroorotic acid (5-FOA). The strain with *Δpyr*FA, *C. bescii* JWCB005, was obtained as a host strain for gene manipulation (Chung et al. 2013a). In order to create a shuttle vector capable of replicating in both *Escherichia coli* and *C. bescii*, the *pyr*F gene for uracil auxotroph was cloned and inserted into pBAS2 vector (Clausen et al. 2004), which is a small plasmid with a replication origin of the two plasmids in *C. bescii*. The *E. coli/C. bescii* shuttle vector pDCW89 was constructed by linking a low copy replication origin of *E. coli*, PSC101, and apramycin-resistant gene cassette (Apr^R^) to pBAS2 vector (Clausen et al. 2004; Dam et al. 2011). Although a shuttle plasmid is available, there is still another barrier that needs to be addressed to manipulate *C. bescii* genes. The biggest obstacle when transforming foreign DNA for deletion/insertion of a gene is the restriction-modification (R-M) system, which recognizes the difference in DNA methylation when foreign DNA is introduced into the cells, leading to the degradation of the foreign DNA by the restriction system in the strain (Chung et al. 2013b). When the pDCW89 shuttle vector is transformed into the *pyr*F deleted strain by electroporation, the transformation competency is significantly low because *C. bescii* has its own restriction endonuclease, CbeI (Cbe_2438), which was discovered by Chung et al. (2011). CbeI has a HaeIII-like activity and is a type II restriction endonuclease that cleaves unmethylated sequences at 5′-GG/CC-3′ (Chung et al. 2013b; Han et al. 2014). The CbeI activity should be removed in the host strains for successful DNA transformation. CbeI (Cbes_2438) and a neighboring α-class N4-cytosine methyltransferase (M.CbeI, Cbes_2437) were confirmed to be the counterpart of the R-M system in *C. bescii* (Chung et al. 2013b). Treatment of the *E. coli*/C. *bescii* shuttle plasmid DNA with cloned M.CbeI protein resulted in efficient transformation. Chung et al. also reported a *cbeI* deletion (Cbes_2438) and generated a new host strain, *C. bescii* JWCB018 (Δ*pyrAF* Δ*cbeI Δldh*), through homologous recombination. JWCB018 (Δ*pyrAF* Δ*cbeI Δldh*) can be transformed by DNA isolated from *E. coli* without in vitro methylation (Chung et al. 2013b). A brief procedure of gene deletion is described in Fig. 3.

### Enhanced hydrogen production by metabolically engineered *C. bescii*

The ultimate goal of biofuel production is to construct a microbial strain for a consolidated bioprocessing (CBP) organism (Olguin-Maciel et al. 2020), which is an organism capable of producing biofuels, such as alcohol and hydrogen gas, through a one-step process using plant biomass (Fig. 2).

Previous research has shown (Cha et al. 2013a, 2016, 2023; Farkas et al. 2013) that various stains of *C. bescii* were constructed using metabolic engineering techniques to enhance its capabilities as a CBP organism (Cha et al. 2013a). The first engineered *C. bescii* strain, obtained through a newly developed metabolic engineering technique, was a mutant strain with a deletion in the lactate dehydrogenase gene (*ldh*), resulting in the complete absence of lactate production. By removing the lactate production pathway, more carbon should be flowed to acetate and more electron flux carried by NADHs runs to acetate and hydrogen production from pyruvate. The *ldh*-deleted strain of *C. bescii* (JWBC017) was grown on 0.5% switchgrass supplemented as the sole carbon source and showed increased production of acetate and hydrogen but no lactate production (Fig. 1) (Cha et al. 2013a). The *ldh*-deleted strain producing no lactate showed a 6.5% conversion of cellobiose to acetate (9.2 mM) with 105% overall carbon recovery (Cha et al. 2013a). The metabolically engineered strain JWCB017 (*ΔpyrFA, Δldh*) produced more hydrogen (~ 3.4 mol/mol of glucose) compared to wild-type *C. bescii* (~ 1.8 mol/mol of glucose) and *C. saccharolyticus* (~ 2.5 mol/mol of glucose) (de Vrije et al. 2007). This indicates that *C. saccharolyticus* wild type produced more hydrogen than *C. bescii* wild type due to the use of yeast extract in the culture media, which can improve growth and yield.

As mentioned earlier, hydrogenases play a key role in microbial energy metabolism, but the exact nature and function of these enzymes remain unclear. Cha et al. (2016) reported the deletion of a gene cluster called *hypABFCDE*, which encodes the maturation proteins for the *C. bescii* [Ni–Fe] hydrogenase. The resulting mutant strain, JWCB038 (Cha et al. 2016), exhibited slower growth compared to its wild type or parent strain (JWCB005, *ΔpyrFA*) because the main function of the [Ni–Fe] hydrogenase may act as a proton pump generating a proton motive force (PMF) across the cellular membrane for ATP synthesis (Fig. 1). The data (Cha et al. 2016) also indicated that the mutant strain JWCB038 did not exhibit a significant reduction in hydrogen production, suggesting that the [Ni–Fe] hydrogenase may not be the main enzyme involved, and that the bifurcating [Fe–Fe] hydrogenase might be the primary enzyme responsible for hydrogen production in *C. bescii*.

To remove the R-M system in *C. bescii* to facilitate metabolic engineering, a *cbe*I deletion strain was generated (Chung et al. 2013b). However, the function of LDH was also disrupted by a native active transposon at the same time (Cha et al. 2013b). The resulting strain JWCB018 (*ΔpyrFA ΔcbeI Δldh)* (Cha et al. 2013b; Chung et al. 2013b) was grown on 10 g/L cellobiose, 20 g/L Avicel, 10 g/L barley straw, and 10 g/L *Miscanthus* as carbon sources and then compared to the *C. bescii* wild type for hydrogen production (Cha et al. 2023). Because of the interruption of the lactate producing pathway in the mutant strain JWCB018, there was an increase in NADHs carrying electrons, resulting in enhanced H_2_ production. This study showed that JWCB018 exhibited up to 25%, 21%, 33%, and 25% increases in H_2_ production on 1.0% cellobiose, 2% Avicel, 1.0% barley straw, and 1.0% *Miscanthus*, respectively (Cha et al. 2023). These findings clearly indicate that appropriate metabolic engineering can significantly enhance the production of H_2_ and other valuable chemicals.

## Further strategies to improve hydrogen production by *C. bescii*

Although very useful gene manipulation techniques for *C. bescii* metabolic engineering have been developed and research for biofuel production is being intensively conducted, there is still a need for the development of more efficient genetic tools and techniques for thermophiles, especially *C. bescii*. For example, promoters for high expression and better replicating plasmids for heterologous gene expression should be developed. Instead of using an uracil auxotroph, new thermo-stable antibiotic screening techniques, such as thermo-stable kanamycin (Lipscomb et al. 2016), will also be needed to save time and effort. However, the current methods for deletion and insertion of multiple genes are sufficient.

There is one possible strategy to increase hydrogen production from real-world plant biomass. This strategy involves utilizing the genetic tools and the techniques developed for *C. bescii*. The first step is to remove the pathway for acetate production by deleting the phosphate acetyltransferase-encoding gene (*pta*, Cbes_1494). By deleting *pta*, the electrons carried by ferredoxins can be used by both types of hydrogenases such as [Fe–Fe] hydrogenases and [Ni–Fe] hydrogenases, resulting in more hydrogen production and ATP synthesis. Another potential strategy involves the manipulating of various glycosyl hydrolases by encoding genes by overexpressing the corresponding genes and manipulating regulatory genes to increase the decomposing efficiency of unpretreated plant biomass.

## Conclusions

In this review, we highlighted that *C. bescii* can produce hydrogen directly from plant biomass without conventional pretreatment processes. Additionally, novel efficient methods for genetic modification of *C. bescii* have been developed through the deletion of *cbeI*, which is a thermostable type II restriction endonuclease. Overall, previous studies have demonstrated that *C. bescii* can be metabolically engineered to enhance hydrogen production. These would help *C. bescii* to efficiently produce hydrogen from biomass and biowaste including lignocellulosic biomass, cattle manure, and wastewater sludge (Table 2).

## References

[CR1] Akhlaghi N, Najafpour-Darzi G (2020) A comprehensive review on biological hydrogen production. Int J Hydrogen Energy 45(43):22492–22512. 10.1016/j.ijhydene.2020.06.182

[CR2] Alfano M, Cavazza C (2020) Structure, function, and biosynthesis of nickel-dependent enzymes. Protein Sci 29(5):1071–1089. 10.1002/pro.3836 32022353 10.1002/pro.3836 PMC7184782

[CR3] Alicia Benitez CW, Andreas de Palmenaer, Michael Lengersdorf, Tim Röding, Thomas Grube, Martin Robinius, Detlef Stolten, Wilhelm Kuckshinrichs (2021) Ecological assessment of fuel cell electric vehicles with special focus on type IV carbon fiber hydrogen tank. J Clean Prod 278 10.1016/j.jclepro.2020.123277

[CR4] Beckers L, Julien Masset C, Hamilton F Delvigne, Toye D, Crine M, Thonart P, Hiligsmann S (2015) Investigation of the links between mass transfer conditions, dissolved hydrogen concentration and biohydrogen production by the pure strain *Clostridium butyricum* CWBI1009. Biochem Eng J 98:18–28. 10.1016/j.bej.2015.01.008

[CR5] Boeke JD, LaCroute F, Fink GR (1984) A positive selection for mutants lacking orotidine-5′-phosphate decarboxylase activity in yeast: 5-fluoro-orotic acid resistance. Mol Gen Genet 197(2):345–6. 10.1007/BF00330984 6394957 10.1007/BF00330984

[CR6] Buckel W (2021) Energy conservation in fermentations of anaerobic bacteria. Front Microbiol 12:703525. 10.3389/fmicb.2021.703525 34589068 10.3389/fmicb.2021.703525 PMC8473912

[CR7] Burkhart BW, Febvre HP, Santangelo TJ (2019) Distinct physiological roles of the three ferredoxins encoded in the hyperthermophilic archaeon *Thermococcus kodakarensis*. mBio 10(2) 10.1128/mBio.02807-18 10.1128/mBio.02807-18 PMC6401487 30837343

[CR8] Cha M, Chung D, Elkins JG, Guss AM, Westpheling J (2013a) Metabolic engineering of *Caldicellulosiruptor bescii* yields increased hydrogen production from lignocellulosic biomass. Biotechnol Biofuels 6(1):85. 10.1186/1754-6834-6-85 23731756 10.1186/1754-6834-6-85 PMC3677179

[CR9] Cha M, Wang H, Chung D, Bennetzen JL, Westpheling J (2013b) Isolation and bioinformatic analysis of a novel transposable element, ISCbe4, from the hyperthermophilic bacterium, *Caldicellulosiruptor**bescii*. J Ind Microbiol Biotechnol 40(12):1443–1448. 10.1007/s10295-013-1345-8 24081709 10.1007/s10295-013-1345-8

[CR10] Cha M, Chung D, Westpheling J (2016) Deletion of a gene cluster for [Ni-Fe] hydrogenase maturation in the anaerobic hyperthermophilic bacterium *Caldicellulosiruptor bescii* identifies its role in hydrogen metabolism. Appl Microbiol Biotechnol 100(4):1823–1831. 10.1007/s00253-015-7025-z 26536872 10.1007/s00253-015-7025-z

[CR11] Cha M, Kim JH, Choi HJ, Nho SB, Kim SY, Cha YL, Song H, Lee WH, Kim SK, Kim SJ (2023) Hydrogen production from barley straw and Miscanthus by the hyperthermophilic bacterium, *Cadicellulosirupter**bescii*. J Microbiol Biotechnol 33(10):1–6. 10.4014/jmb.2305.05022 37463861 10.4014/jmb.2305.05022 PMC10619549

[CR12] Chandel NS (2021) Glycolysis. Cold Spring Harbor Perspect Biol 13(5):a040535 10.1101/cshperspect.a040535 PMC8091952 33941515

[CR13] Chung DH, Huddleston JR, Farkas J, Westpheling J (2011) Identification and characterization of CbeI, a novel thermostable restriction enzyme from *Caldicellulosiruptor bescii* DSM 6725 and a member of a new subfamily of HaeIII-like enzymes. J Ind Microbiol Biotechnol 38(11):1867–1877. 10.1007/s10295-011-0976-x 21604181 10.1007/s10295-011-0976-x PMC4269323

[CR14] Chung D, Cha M, Farkas J, Westpheling J (2013a) Construction of a stable replicating shuttle vector for *Caldicellulosiruptor* species: use for extending genetic methodologies to other members of this genus. PLoS One 8(5):e62881. 10.1371/journal.pone.0062881 23658781 10.1371/journal.pone.0062881 PMC3643907

[CR15] Chung D, Farkas J, Westpheling J (2013b) Overcoming restriction as a barrier to DNA transformation in *Caldicellulosiruptor* species results in efficient marker replacement. Biotechnol Biofuels 6(1):82. 10.1186/1754-6834-6-82 23714229 10.1186/1754-6834-6-82 PMC3679861

[CR16] Chung D, Cha M, Guss AM, Westpheling J (2014) Direct conversion of plant biomass to ethanol by engineered *Caldicellulosiruptor bescii*. Proc Natl Acad Sci USA 111(24):8931–8936. 10.1073/pnas.1402210111 24889625 10.1073/pnas.1402210111 PMC4066518

[CR17] Clausen A, Mikkelsen MJ, Schroder I, Ahring BK (2004) Cloning, sequencing, and sequence analysis of two novel plasmids from the thermophilic anaerobic bacterium *Anaerocellum thermophilum*. Plasmid 52(2):131–138. 10.1016/j.plasmid.2004.06.001 15336490 10.1016/j.plasmid.2004.06.001

[CR18] Cofré O, Ramirez M, Gómez JM, Cantero D (2016) Pilot scale fed-batch fermentation in a closed loop mixed reactor for the biotransformation of crude glycerol into ethanol and hydrogen by *Escherichia coli* MG1655. Biomass Bioenergy 91:37–47. 10.1016/j.biombioe.2016.04.015

[CR19] Crosby JR, Laemthong T, Lewis AM, Straub CT, Adams MWW, Kelly RM (2019) Extreme thermophiles as emerging metabolic engineering platforms. Curr Opin Biotechnol 59:55–64. 10.1016/j.copbio.2019.02.006 30875665 10.1016/j.copbio.2019.02.006

[CR20] Dam P, Kataeva I, Yang SJ, Zhou F, Yin Y, Chou W, Poole FL 2nd, Westpheling J, Hettich R, Giannone R, Lewis DL, Kelly R, Gilbert HJ, Henrissat B, Xu Y, Adams MW (2011) Insights into plant biomass conversion from the genome of the anaerobic thermophilic bacterium *Caldicellulosiruptor bescii* DSM 6725. Nucleic Acids Res 39(8):3240–3254. 10.1093/nar/gkq1281 21227922 10.1093/nar/gkq1281 PMC3082886

[CR21] David B, Levin RI, Cicek N, Sparling R (2006) Hydrogen production by *Clostridium thermocellum* 27405 from cellulosic biomass substrates. Int J Hydrogen Energy 31:1496–1503. 10.1016/j.ijhydene.2006.06.015

[CR22] de Vrije T, Mars AE, Budde MA, Lai MH, Dijkema C, de Waard P, Claassen PA (2007) Glycolytic pathway and hydrogen yield studies of the extreme thermophile *Caldicellulosiruptor saccharolyticus*. Appl Microbiol Biotechnol 74(6):1358–1367. 10.1007/s00253-006-0783-x 17216445 10.1007/s00253-006-0783-x

[CR23] de Vrije T, Bude MAW, Lips SJ, Bakker RR, Mars AE, Claassen PAM (2010) Hydrogen production from carrot pulp by the extreme thermophiles *Caldicellulosiruptor saccharolyticus* and *Thermotoga neapolitana*. Int J Hydrogen Energy 35(24):13206–13213. 10.1016/j.ijhydene.2010.09.014

[CR24] Esercizio N, Lanzilli M, Vastano M, Landi S, Xu Z, Gallo C, Nuzzo G, Manzo E, Fontana A, d’Ippolito G (2021) Fermentation of biodegradable organic waste by the family *Thermotogaceae*. Resources 10(4):34. 10.3390/resources10040034

[CR25] Farkas J, Chung D, Cha M, Copeland J, Grayeski P, Westpheling J (2013) Improved growth media and culture techniques for genetic analysis and assessment of biomass utilization by *Caldicellulosiruptor bescii*. J Ind Microbiol Biotechnol 40(1):41–49. 10.1007/s10295-012-1202-1 23149625 10.1007/s10295-012-1202-1 PMC4290016

[CR26] Fawzy S, Osman AI, Doran J, Rooney DW (2020) Strategies for mitigation of climate change: a review. Environ Chem Lett 18:2069–2094. 10.1007/s10311-020-01059-w

[CR27] Ghimire A, Frunzo L, Pontoni L, d’Antonio G, Lens PN, Esposito G, Pirozzi F (2015) Dark fermentation of complex waste biomass for biohydrogen production by pretreated thermophilic anaerobic digestate. J Environ Manage 152:43–48. 10.1016/j.jenvman.2014.12.049 25617867 10.1016/j.jenvman.2014.12.049

[CR28] Han D, Xu Z (2017) Development of a pyrE-based selective system for *Thermotoga* sp. strain RQ7. Extremophiles 21(2):297–306. 10.1007/s00792-016-0902-2 27928679 10.1007/s00792-016-0902-2

[CR29] Han D, Xu H, Puranik R, Xu Z (2014) Natural transformation of *Thermotoga* sp. strain RQ7. BMC Biotechnol 14:39. 10.1186/1472-6750-14-39 24884561 10.1186/1472-6750-14-39 PMC4029938

[CR30] Hassan Q, Sameen AZ, Salman HM, Jaszczur M, Al-Jiboory AK (2023) Hydrogen energy future: advancements in storage technologies and implications for sustainability. J Energy Storage 72:108404. 10.1016/j.est.2023.108404

[CR31] Hoang AT, Sirohi R, Pandey A, Nižetić S, Lam SS, Chen W-H, Luque R, Thomas S, Arıcı M, Pham VV (2023) Biofuel production from microalgae: challenges and chances. Phytochem Rev 22:1089–1126. 10.1007/s11101-022-09819-y

[CR32] Jay ZJ, Hunt KA, Chou KJ, Schut GJ, Maness PC, Adams MWW, Carlson RP (2020) Integrated thermodynamic analysis of electron bifurcating [FeFe]-hydrogenase to inform anaerobic metabolism and H(2) production. Biochim Biophys Acta Bioenerg 1:148087. 10.1016/j.bbabio.2019.148087 10.1016/j.bbabio.2019.148087 31669490

[CR33] Jayasinghearachchi HS, Sarma PM, Singh S, Aginihotri A, Mandal AK, Lal B (2009) Fermentative hydrogen production by two novel strains of *Enterobacter aerogenes* HGN-2 and HT 34 isolated from sea buried crude oil pipelines. Int J Hydrogen Energy 34(17):7197–7207. 10.1016/j.ijhydene.2009.06.079

[CR34] Jeswani HK, Chilvers A, Azapagic A (2020) Environmental sustainability of biofuels: a review. Proc Math Phys Eng Sci 476(2243):20200351. 10.1098/rspa.2020.0351 33363439 10.1098/rspa.2020.0351 PMC7735313

[CR35] Kaila VRI, Wikstrom M (2021) Architecture of bacterial respiratory chains. Nat Rev Microbiol 19(5):319–330. 10.1038/s41579-020-00486-4 33437024 10.1038/s41579-020-00486-4

[CR36] Kanai T, Imanaka H, Nakajima A, Uwamori K, Omori Y, Fukui T, Atomi H, Imanaka T (2005) Continuous hydrogen production by the hyperthermophilic archaeon, *Thermococcus kodakaraensis* KOD1. J Biotechnol 116(3):271–282. 10.1016/j.jbiotec.2004.11.002 15707688 10.1016/j.jbiotec.2004.11.002

[CR37] Kim SK, Chung D, Himmel ME, Bomble YJ, Westpheling J (2019) Heterologous co-expression of two beta-glucanases and a cellobiose phosphorylase resulted in a significant increase in the cellulolytic activity of *the Caldicellulosiruptor bescii* exoproteome. J Ind Microbiol Biotechnol 46(5):687–695. 10.1007/s10295-019-02150-0 30783893 10.1007/s10295-019-02150-0

[CR38] Kim J, Hwang S, Lee SM (2022) Metabolic engineering for the utilization of carbohydrate portions of lignocellulosic biomass. Metab Eng 71:2–12. 10.1016/j.ymben.2021.10.002 34626808 10.1016/j.ymben.2021.10.002

[CR39] Lepage T, Kammoun M, Schmetz Q, Richel A (2021) Biomass-to-hydrogen: a review of main routes production, processes evaluation and techno-economical assessment. Biomass Bioenergy 144:105920. 10.1016/j.biombioe.2020.105920

[CR40] Lin P-Y, Whang L-M, Wu Y-R, Ren W-J, Hsiao C-J, Li S-L, Chang J-S (2007) Biological hydrogen production of the genus *Clostridium*: metabolic study and mathematical model simulation. Int J Hydrogen Energy 32(12):1728–1735. 10.1016/j.ijhydene.2006.12.009

[CR41] Lin C-Y, Chang C-C, Hung C-H (2008) Fermentative hydrogen production from starch using natural mixed cultures. Int J Hydrogen Energy 33:2445–2453. 10.1016/j.ijhydene.2008.02.069

[CR42] Lipscomb GL, Conway JM, Blumer-Schuette SE, Kelly RM, Adams MWW (2016) A highly thermostable kanamycin resistance marker expands the tool kit for genetic manipulation of *Caldicellulosiruptor bescii*. Appl Environ Microbiol 82(14):4421–4428. 10.1128/AEM.00570-16 27208106 10.1128/AEM.00570-16 PMC4959222

[CR43] Liu X, Zhu Y, Yang ST (2006) Construction and characterization of ack deleted mutant of *Clostridium tyrobutyricum* for enhanced butyric acid and hydrogen production. Biotechnol Prog 22(5):1265–1275. 10.1021/bp060082g 17022663 10.1021/bp060082g

[CR44] Lu Y, Koo J (2019) O(2) sensitivity and H(2) production activity of hydrogenases-a review. Biotechnol Bioeng 116(11):3124–3135. 10.1002/bit.27136 31403182 10.1002/bit.27136

[CR45] Lucas S, Toffin L, Zivanovic Y, Charlier D, Moussard H, Forterre P, Prieur D, Erauso G (2002) Construction of a shuttle vector for, and spheroplast transformation of, the hyperthermophilic archaeon *Pyrococcus abyssi*. Appl Environ Microbiol 68(11):5528–5536. 10.1128/AEM.68.11.5528-5536.2002 12406746 10.1128/AEM.68.11.5528-5536.2002 PMC129897

[CR46] Manisalidis I, Stavropoulou E, Stavropoulos A, Bezirtzoglou E (2020) Environmental and health impacts of air pollution: a review. Front Public Health 8:14. 10.3389/fpubh.2020.00014 32154200 10.3389/fpubh.2020.00014 PMC7044178

[CR47] Martínez-Jaramillo JE, Arango-Aramburo S, Giraldo-Ramírez DP (2019) The effects of biofuels on food security: a system dynamics approach for the Colombian case. Sustain Energy Technol Assess 34:97–109. 10.1016/j.seta.2019.05.009

[CR48] Martins F, Felgueiras C, Smitkova M, Caetano N (2019) Analysis of fossil fuel energy consumption and environmental impacts in European countries. Energies 12(6):964. 10.3390/en12060964

[CR49] Miller MA, Petrasch J, Randhir K, Rahmatian N, Klausner J (2021) Thermal, mechanical, and hybrid chemical energy storage systems/chapter 5 - chemical energy storage. Acad Press. 10.1016/C2019-0-00430-X

[CR50] Minnan L, Jinli H, Xiaobin W, Huijuan X, Jinzao C, Chuannan L, Fengzhang Z, Liangshu X (2005) Isolation and characterization of a high H2-producing strain *Klebsiella oxytoca* HP1 from a hot spring. Res Microbiol 156(1):76–81. 10.1016/j.resmic.2004.08.004 15636750 10.1016/j.resmic.2004.08.004

[CR51] Nanqi R, Wanqian G, Bingfeng L, Guangli C, Jie D (2011) Biological hydrogen production by dark fermentation: challenges and prospects towards scaled-up production. Curr Opin Biotechnol 22(3):365–70. 10.1016/j.copbio.2011.04.022 21612910 10.1016/j.copbio.2011.04.022

[CR52] Niu K, Zhang X, Tan W-S, Zhu M-L (2010) Characteristics of fermentative hydrogen production with *Klebsiella pneumoniae* ECU-15 isolated from anaerobic sewage sludge. Int J Hydrogen Energy 35(1):71–80. 10.1016/j.ijhydene.2009.10.071

[CR53] Oh S-E, Zuo Y, Zhang H, Guiltinan MJ, Logan BE, Regan JM (2009) Hydrogen production by *Clostridium acetobutylicum* ATCC824 and megaplasmid-deficient mutant M5 evaluated usinga large headspace volume technique. Int J Hydrogen Energy 34:9347–9353. 10.1016/j.ijhydene.2009.09.084

[CR54] Okolie JA, Patra BR, Mukherjee A, Nanda S, Dalai AK, Kozinski JA (2021) Futuristic applications of hydrogen in energy, biorefining, aerospace, pharmaceuticals and metallurgy. Int J Hydrogen Energy 46:8885–8905. 10.1016/j.ijhydene.2021.01.014

[CR55] Olguin-Maciel E, Singh A, Chable-Villacis R, Tapia-Tussell R, Ruiz HA (2020) Consolidated bioprocessing, an innovative strategy towards sustainability for biofuels production from crop residues: an overview. Agronomy 10:1834. 10.3390/agronomy10111834

[CR56] Palone O, Gagliardi GG, Mechelli M, Cedola L, Borello D (2023) Techno-economic analysis of sustainable methanol and ammonia production by chemical looping hydrogen generation from waste plastic. Energy Convers Manag 292:117389. 10.1016/j.enconman.2023.117389

[CR57] Periyasamy S, Isabel B, Kavitha S, Karthik D, Mohamed BA, Gizaw DG, Sivashanmugam P, Aminabhavi TM (2023) Recent advances in consolidated bioprocessing for conversion of lignocellulosic biomass into bioethanol – a review. Chem Eng J 453:139783. 10.1016/j.cej.2022.139783

[CR58] Schroder C, Selig M, Schonheit P (1994) Glucose fermentation to acetate, CO2 and H 2 in the anaerobic hyperthermophilic eubacterium *Thermotoga maritima*: involvement of the Embden-Meyerhof pathway. Arch Microbiol 161:460–470. 10.1007/BF00307766

[CR59] Schut GJ, Adams MW (2009) The iron-hydrogenase of *Thermotoga maritima* utilizes ferredoxin and NADH synergistically: a new perspective on anaerobic hydrogen production. J Bacteriol 191(13):4451–4457. 10.1128/JB.01582-08 19411328 10.1128/JB.01582-08 PMC2698477

[CR60] Scott IM, Rubinstein GM, Poole FL 2nd, Lipscomb GL, Schut GJ, Williams-Rhaesa AM, Stevenson DM, Amador-Noguez D, Kelly RM, Adams MWW (2019) The thermophilic biomass-degrading bacterium *Caldicellulosiruptor bescii* utilizes two enzymes to oxidize glyceraldehyde 3-phosphate during glycolysis. J Biol Chem 294(25):9995–10005. 10.1074/jbc.RA111.007120 31097544 10.1074/jbc.RA118.007120 PMC6597818

[CR61] Seppälä JJ, Puhakka JA, Yli-Harja O, Karp MT, Santala V (2011) Fermentative hydrogen production by Clostridium butyricum and *Escherichia coli* in pure and cocultures. Int J Hydrogen Energy 36(17):10701–10708

[CR62] Servé WM, Kengen AJMS (1994) Growth and energy conservation in batch cultures of *Pyrococcus furiosus*. FEMS Microbiol Lett 117:305–309. 10.1111/j.1574-6968.1994.tb06784.x

[CR63] Shahbeik H, Peng W, Panahi HKS, Dehhaghi M, Guillemin GJ, Fallahi A, Amiri H, Rehan M, Raikwar D, Latine H, Pandalone B, Khoshnevisan B, Sonne C, Vaccaro L, Nizami A-S, Gupta VK, Lam SS, Pan J, Luque R, Sels B, Tabatabaei M, Aghbashlo M (2022) Synthesis of liquid biofuels from biomass by hydrothermal gasification: a critical review. Renew Sust Energ Rev 167:112833. 10.1016/j.rser.2022.112833

[CR64] Singh R, Tevatia R, White D, Demirel Y, Blum P (2019) Comparative kinetic modeling of growth and molecular hydrogen overproduction by engineered strains of *Thermotoga maritima*. Int J Hydrogen Energy 44:7125–7136. 10.1016/j.ijhydene.2019.01.124

[CR65] Soboh B, Linder D, Hedderich R (2004) A multisubunit membrane-bound [NiFe] hydrogenase and an NADH-dependent Fe-only hydrogenase in the fermenting bacterium *Thermoanaerobacter tengcongensis*. Microbiology (reading) 150(Pt 7):2451–2463. 10.1099/mic.0.27159-0 15256587 10.1099/mic.0.27159-0

[CR66] Song Y, Liu M, Xie L, You C, Sun J, Zhang YPJ (2019) A recombinant 12-His tagged Pyrococcus furiosus soluble [NiFe]-hydrogenase I overexpressed in *Thermococcus kodakarensis* KOD1 facilitates hydrogen-powered in vitro NADH regeneration. Biotechnol J 14(4):e1800301. 10.1002/biot.201800301 30307115 10.1002/biot.201800301

[CR67] Straub CT, Bing RG, Otten JK, Keller LM, Zeldes BM, Adams MWW, Kelly RM (2020) Metabolically engineered *Caldicellulosiruptor bescii* as a platform for producing acetone and hydrogen from lignocellulose. Biotechnol Bioeng 117(12):3799–3808. 10.1002/bit.27529 32770740 10.1002/bit.27529 PMC11719096

[CR68] Tanisho S (1998) Hydrogen production by facultative anaerobe *Enterobacter aerogenes*, BioHydrogen. Springer, Boston, MA 10.1007/978-0-585-35132-2_35

[CR69] Vancanneyt M, De Vos P, Maras M, De Ley J (1990) Ethanol production in batch and continuous culture from some carbohydrates with *Clostridium thermosaccharolyticum* LMG 656. Syst Appl Microbiol 13:382–387. 10.1016/S0723-2020(11)80237-7

[CR70] Wagner M, van Wolferen M, Wagner A, Lassak K, Meyer BH, Reimann J, Albers SV (2012) Versatile genetic tool box for the crenarchaeote *Sulfolobus acidocaldarius*. Front Microbiol 3:214. 10.3389/fmicb.2012.00214 22707949 10.3389/fmicb.2012.00214 PMC3374326

[CR71] White D (2012) The physiology and biochemistry of prokaryotes. Oxford University Press

[CR72] Willquist K, Zeidan AA, van Niel EW (2010) Physiological characteristics of the extreme thermophile *Caldicellulosiruptor saccharolyticus*: an efficient hydrogen cell factory. Microb Cell Fact 9:89. 10.1186/1475-2859-9-89 21092203 10.1186/1475-2859-9-89 PMC3003633

[CR73] Yan Liu PY, Song X, Yinbo Qu (2008) Hydrogen production from cellulose by co-culture of *Clostridium thermocellum* JN4 and *Thermoanaerobacterium thermosaccharolyticum* GD17. Int J Hydrogen Energy 33(12):2927–2933. 10.1016/j.ijhydene.2008.04.004

[CR74] Yilmazel YD, Duran M (2021) Biohydrogen production from cattle manure and its mixtures with renewable feedstock by hyperthermophilic *Caldicellulosiruptor bescii*. J Clea Prod 292:125969. 10.1016/j.jclepro.2021.125969

[CR75] Yoshida A, Nishimura T, Kawaguchi H, Inui M, Yukawa H (2006) Enhanced hydrogen production from glucose using ldh- and frd-inactivated *Escherichia coli* strains. Appl Microbiol Biotechnol 73(1):67–72. 10.1007/s00253-006-0456-9 16683133 10.1007/s00253-006-0456-9

[CR76] Zafar A, Aftab MN, Asif A, Karadag A, Peng L, Celebioglu HU, Afzal MS, Hamid A, Iqbal I (2021) Efficient biomass saccharification using a novel cellobiohydrolase from *Clostridium clariflavum* for utilization in biofuel industry. RSC Adv 11(16):9246–9261. 10.1039/d1ra00545f 35423428 10.1039/d1ra00545f PMC8695235

[CR77] Zhang K, Zhao W, Rodionov DA, Rubinstein GM, Nguyen DN, Tanwee TNN, Crosby J, Bing RG, Kelly RM, Adams MWW, Zhang Y (2021) Genome-scale metabolic model of *Caldicellulosiruptor bescii* reveals optimal metabolic engineering strategies for bio-based chemical production. mSystems 6(3):e0135120. 10.1128/mSystems.01351-20 34060912 10.1128/mSystems.01351-20 PMC8269263

[CR78] Zheng T, Huang Q, Zhang C, Ni J, She Q, Shen Y (2012) Development of a simvastatin selection marker for a hyperthermophilic acidophile, *Sulfolobus**islandicus*. Appl Environ Microbiol 78(2):568–574. 10.1128/AEM.06095-11 22081574 10.1128/AEM.06095-11 PMC3255719

